# Sit‐to‐stand and stand‐to‐sit kinematics in older adults with and without functional disability: A principal component analysis

**DOI:** 10.1111/ajag.70089

**Published:** 2025-09-02

**Authors:** Juliana Moreira, Bruno Cunha, José Félix, Rubim Santos, Andreia S. P. Sousa

**Affiliations:** ^1^ CIR, E2S, Polytechnic of Porto Porto Portugal; ^2^ Research Center in Physical Activity, Health and Leisure, Faculty of Sports University of Porto Porto Portugal; ^3^ CINTESIS@Rise, CINTESIS.UPT Portucalense University Porto Portugal; ^4^ Department of Physiotherapy, Institute of Health of the North – Escola Superior de Saúde do Vale do Ave (ESSVA) Cooperativa de Ensino Superior Politécnico e Universitário (CESPU) Vila Nova de Famalicão Portugal; ^5^ Department of Medical Sciences University of Aveiro Aveiro Portugal

**Keywords:** Activities of Daily Living, Biomechanical Phenomena, Factor Analysis, Statistical, Geriatrics

## Abstract

**Objective:**

Sit‐to‐Stand (Sit‐TS) and Stand‐to‐Sit (Stand‐TS) transitions are essential daily movements affected by ageing and disability. This study aimed to explore related kinematic domains in older adults with and without disability.

**Methods:**

A cross‐sectional study including adults aged 60 years or older, with (*n* = 25) and without disability (*n* = 35). Comparisons between groups included task time, centre of mass (CoM) acceleration, postural sway and principal component (PC) scores for each task. Principal component models (PCMs) included lower limb and trunk tridimensional joint ranges of motion, angular velocity range, CoM displacement and velocity along each Sit‐TS (flexion, momentum transfer, extension and stabilisation) and Stand‐TS (initiation, flexion, momentum transfer and extension) phases.

**Results:**

Older adults with functional disability exhibited increased Sit‐TS peak antero‐posterior CoM acceleration (*p* = .02). The Sit‐TS and Stand‐TS PCMs included nine PCs each. In Sit‐TS, the first three explained half the variance: PC1 captured transverse hip and knee stabilisation kinematics, PC2 described trunk and hip frontal and transverse control during flexion, and PC3 represented sagittal knee and ankle control during momentum transfer and extension. In Stand‐TS, variance was more distributed (PC1 describing frontal hip and knee flexion velocity, PC2 sagittal trunk and hip extension velocity, and PC3 vertical CoM velocity at extension). Significant group differences emerged in PC4 (transverse knee and frontal hip kinematics) and PC9 (sagittal and frontal trunk angular velocity ranges during momentum transfer).

**Conclusions:**

Both transitions revealed distinct joint and trunk control demands. Principal components involving transverse knee, frontal hip and trunk angular velocities distinguished disability groups, with Stand‐TS showing greater discriminative power.

**Practice impact**

Principal component analysis reveals that Stand‐To‐Sit movements are more discriminative of functional disability than Sit‐to‐Stand. This suggests that assessing Stand‐To‐Sit kinematics, particularly hip and knee flexion velocity and Centre‐of‐Mass vertical velocity, could serve as an early screening tool for detecting functional decline in older adults before more severe impairments develop.

## 1 INTRODUCTION

Sit‐to‐stand (Sit‐TS) and stand‐to‐sit (Stand‐TS) demand coordinated bilateral lower limb and trunk movements to raise the body's centre of mass (CoM), while maintaining postural stability.^1^, ^2^ Age‐related alterations render older adults increasingly challenged by the transition from sitting to standing.^1^, ^3^ These transitions are essential components of daily functioning, often repeated throughout the day.^4^ Their successful execution reflects lower‐limb strength, balance control and neuromuscular coordination, all of which are critical for maintaining autonomy.^1^, ^2^

A recent systematic review exploring biomechanical and neuromuscular control characteristics of Sit‐TS transition in young and older adults reported that older adults showed slower transfer time, increased trunk flexion and higher horizontal body sway.^2^ Complementary research has applied principal component analysis (PCA) to Sit‐TS, analysing kinematic time series data with and without hand support. Prior findings showed reduced trunk movement in older adults performing Sit‐TS without hand support, though PCA revealed no significant differences in angular velocity components.^5^ PCA can also be used on discrete kinematic variables to reduce data dimensionality and uncover underlying patterns of movement coordination. This approach is particularly useful in ageing populations, where high inter‐individual variability and compensatory strategies may obscure consistent group‐level trends. Unlike traditional multivariate statistical techniques, PCA enables the transformation of correlated input variables into a smaller set of uncorrelated components,^6^, ^7^ facilitating the identification of the most influential biomechanical features.

Previous studies have demonstrated that older adults with functional limitations exhibit altered Sit‐TS and Stand‐TS strategies, including slower time, reduced joint ranges of motion (ROM) and increased reliance on compensatory strategies, such as altered trunk posture or asymmetrical loading.^8^ These adaptations may reflect underlying neuromuscular impairments and contribute to reduced mobility and increased fall risk in populations with disabilities.

While ageing movement‐related adaptations have been described for Sit‐TS transition, the Stand‐TS transition has been less explored. Moreover, the association between age‐related adaptations and disability remains poorly investigated. Thus, the aim of this study was to explore the kinematics of Sit‐TS and Stand‐TS transitions among older adults and investigate the differences in the domains between those with and without functional disability.

## 2 METHODS

### 2.1 Study design and participants

This was a cross‐sectional study conducted in accordance with Strengthening the Reporting of Observational Studies in Epidemiology^9^ guidelines, and integrated within a larger study whose protocol is registered in the ClinicalTrials.gov database (register identifier: NCT05611723).

The study sample included older adults, 60 years or older, living in the community and able to perform Sit‐TS and Stand‐TS independently. Volunteers were excluded from the study if they were institutionalised or had conditions that influenced the performance of tasks.

Once enrolled in the study, participants were divided into two groups: older adults without disabilities and those exhibiting two or more disability indicators identified in previous research.^10^ These indicators included handgrip strength (HGS), balance, overall self‐reported health status (SRH), basic activities of daily living (ADL) and instrumental activities of daily living (IADL).

### 2.2 Instruments

The demographic data, health conditions, prescribed medication, history of falls within the previous 12 months, Mini Mental State Examination (MMSE) and seven‐item short version of the International Physical Activity Questionnaire (IPAQ) were collected through a questionnaire. The disability assessment tools included the Barthel Index of ADL, the Lawton and Brody IADL Scale and SRH.^10^ Handgrip strength and the One‐Leg Standing Test (OLST) were used to further evaluate disability.^10^ The Barthel Index scoring ranges from 0, indicating complete dependency, to 20, indicating functional independence.^11^ On the Lawton and Brody IADL scale, participants are classified as globally dependent if they exhibit impairment in at least one IADL item.^12^, ^13^ Self‐reported health was evaluated using the question: ‘In general, how do you rate your health today? Very bad, Bad, Fair, Good, Very Good’. For analysis, SRH was dichotomised into good (‘very good’ or ‘good’) or poor SRH (‘fair’, ‘bad’, ‘very bad’).^14^, ^15^ The HGS was measured with a Jamar® Plus+ Digital dynamometer (Performance Health Supply, Cedarburg, WI, USA), following the protocol recommended by the American Society of Hand Therapists.^16^, ^17^ The European Working Group on Sarcopenia in Older People 2 established cut‐off values to identify sarcopenia (<27 kg for men and <16 kg for women). On the performance of OLST, the ability to successfully complete the 10 s is independently associated with all‐cause mortality.^18^

The definition of disability is grounded in the International Classification of Functioning, Disability and Health framework.^19^ However, since indicators related to environmental factors and body structures were not assessed, we adopted the term *functional disability*,^20^ reflecting a focus on limitations primarily in body functions, activities and participation domains.

The kinematic and kinetic data were collected synchronously, using the Qualisys Track Manager (Qualisys AB®, Sweden) with 12 optoelectronic cameras (eight Oquos500, four MiqusM3) and one Miqus video camera at an acquisition rate of 100 Hz. An 81 reflective marker set‐up was used.^21^

### 2.3 Procedures and data collection

The kinematic data collection procedure included the performance of tasks using an armless chair .46 metres in height. Participants with comfortable and usual footwear, with no heels, were instructed to stand up from the chair at a self‐selected speed and with their arms folded across the chest. After reaching the upright standing position, participants stood still for 3 s and then sat down again.^3^, ^22^ Participants were asked to perform the task three times.^22^

### 2.4 Data processing

Each marker trajectory was identified and its accuracy during data collection was verified using the Qualisys Track Manager software (Qualisys AB®, Sweden). The biomechanical full body model was applied in Visual3D Professional (C‐Motion, Inc., EUA), and a 6‐Hz low‐pass Butterworth filter was applied to marker trajectory data. A Cardan sequence was used that assumes that the x axis is in the medial–lateral direction, the y axis is anterior–posterior, and the z axis is in the up and down or axial direction.^23^

Kinematic parameters included the time to perform each task, CoM peak acceleration for Sit‐TS, CoM peak deceleration for Stand‐TS, and anteroposterior (AP) and mediolateral (ML) postural sway both for Sit‐TS and Stand‐TS. The CoM postural sway was measured by the standard deviation of the CoM acceleration.^2^ The trunk, hip, knee and ankle ROM, and range of angular velocity were computed by subtracting the maximum value of each parameter from its minimum for each phase of Sit‐TS and Stand‐TS, defined ahead. The same computation was applied to the CoM position and velocity to calculate the CoM displacement and velocity range. Lower limb and trunk joint angles were measured in degrees and calculated as recommended by the International Society for Biomechanics (ISB) for the lower limb.^23^, ^24^ The joint angular velocities of trunk and lower limb movements were computed in degrees per second, describing the relative angular velocity of one segment relative to another segment.^23^ The CoM was computed for all body segments by considering the length of the body segments from the joint centres, and the position had as a resolution coordinate system the laboratory coordinate system. These variables were included given their relevance to task performance as supported by previous literature.^2^

The Sit‐TS and Stand‐TS tasks were divided into four phases each, according to previous literature on older adult movement analysis (Figure S1).^22^ The Sit‐TS was divided into flexion, momentum transfer, extension and stabilisation.^22^ The Stand‐TS task was divided into initiation, flexion, momentum transfer and extension.^22^

### 2.5 Data analysis

Data analysis was conducted using IBM SPSS Statistics for Macintosh, Version 29.0.2.0, (Armonk, NY: IBM Corp) with a significance level set at .05 for the statistical analysis. Between‐group comparisons were assessed either with Mann–Whitney tests or independent sample *t*‐tests, as appropriate, and *χ* ^2^ tests were employed to analyse associations between‐group for categorical variables, specifically sex, history of falls and SRH status.

Principal component analysis was conducted on the tridimensional lower limb joints and trunk ROM, angular velocity ranges and the CoM displacement and velocity ranges to construct distinct principal component models (PCM) for each phase of Sit‐TS and Stand‐TS transitions. The aim was to reduce dimensionality and identify dominant movement patterns during each phase of Sit‐TS and Stand‐TS transitions. This data‐driven approach allows the detection of latent coordination strategies that may not be captured through traditional multivariate comparisons. Potential outliers were manually inspected in the data, and one severe outlier was identified across multiple kinematic parameters in the Stand‐TS task.

A correlation matrix was used to ensure that each variable contributed equally to the analysis. The suitability of the dataset for PCA was determined using the Kaiser–Meyer–Olkin (KMO) as a measure of sampling adequacy and Bartlett's test of Sphericity, which assesses the appropriateness of data for dimensionality reduction. PCA was deemed suitable if Bartlett's test yielded a statistically significant result (*p* < .05) and KMO values exceeded .5.

Principal components (PCs) were extracted for each PCM phase based on eigenvalues greater than 1, accounting for the majority of the variance. To enhance the interpretability of the components, a Varimax rotation was applied. The resulting rotated component matrix was analysed to identify clusters of variables that loaded onto the same components, with emphasis placed on variables exhibiting high loadings (loading >.8).^25^ Subsequently, a PCM for each task was developed by aggregating these parameters with loadings exceeding .8 for each phase. On Stand‐TS PCM, four variables with communalities (representing the proportion of variance explained by the factors) lower than .650 were excluded to achieve a KMO value greater than .5, reflecting only variables that meaningfully contributed to the extracted components.

### 2.6 Ethics statement

The study adhered rigorously to the principles outlined in the Declaration of Helsinki. All participants received information about the study's purpose and methodology, and completed an informed consent form. The study was submitted to the E‐2S, Polytechnic of Porto Institutional Ethics Committee on 27 April 2022 and obtained authorisation on 25 May 2022 (Approval No CE0064C).

## 3 RESULTS

**FIGURE 1:**
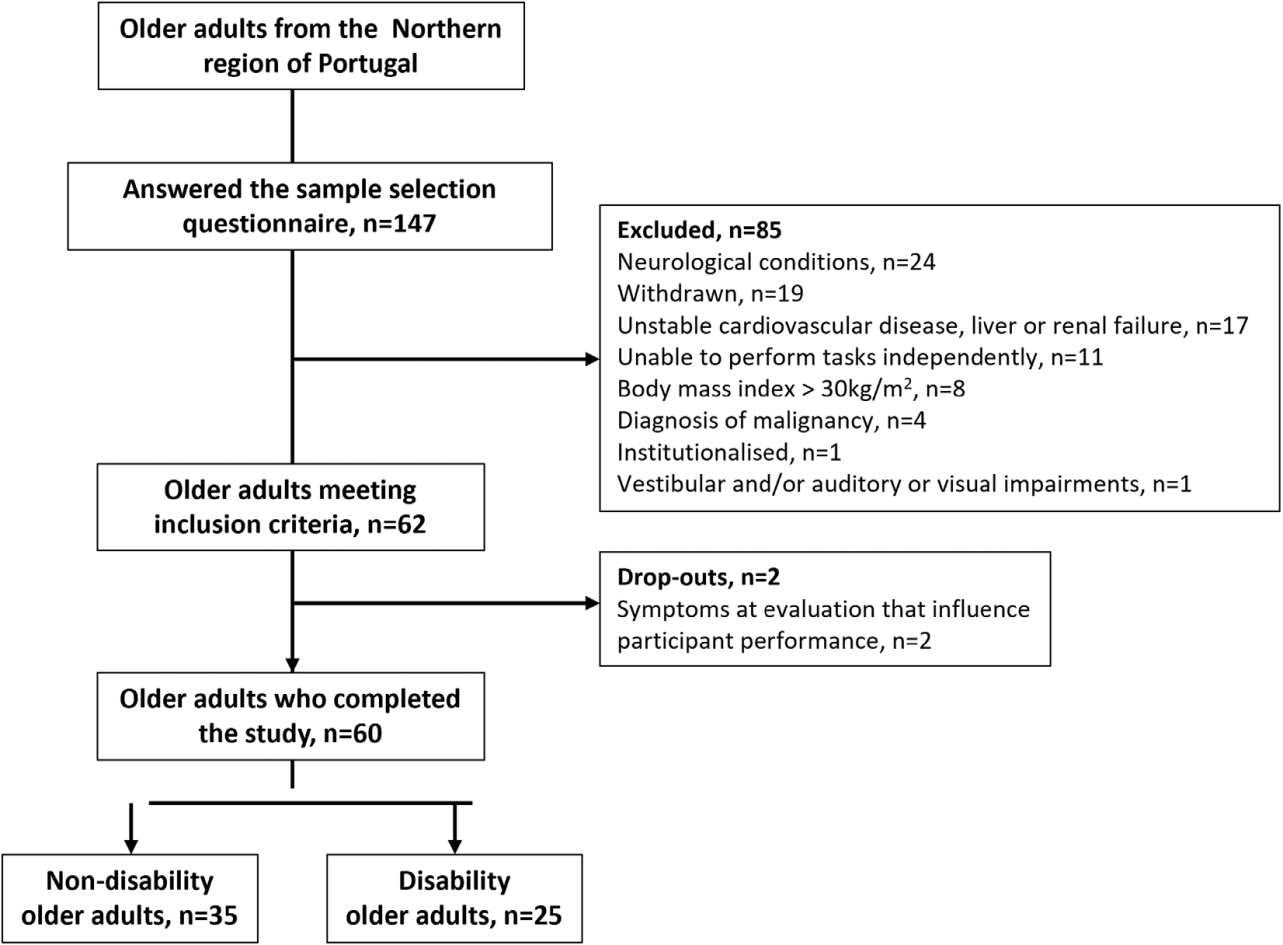
Flowchart illustrating the participant selection process.

A total of 62 participants out of 147 older adults initially participated in the study, as illustrated in Figure 1. The final analysis incorporated data from 60 participants, as two participants were unable to perform the tasks due to symptoms presented during the assessment session. Descriptive statistics summarising demographic and clinical characteristics are presented in Table 1.

**TABLE 1:** Demographic, clinical disability indicators characterisation of the sample.

	OA (*n* = 60)	ND (*n* = 35)	D (*n* = 25)	*p*‐Value (test value)
Demographic and clinical data				
Age, years	67.86 ± 6.46	66.34 ± 5.60	68.60 ± 6.77	.15 (534)^(a)^
Gender (*n* female)	38 (63)	19 (54)	19 (76)	.09 (2.961)^(b)^
BMI (kg/m^2^)	25.39 ± 2.96	25.22 ± 3.08	26.02 ± 2.66	.30 (−1.049)^(c)^
History of fall (*n* fallers)	22 (37)	11 (31)	11 (44)	.47 (.525)^(b)^
Polypharmacy (*n* polymedicated)	13 (22)	2 (6)	11 (44)	**<.001** (12.595)^(b)^
Cognitive function (MMSE score)	28.74 ± 1.38	28.94 ± 1.31	28.68 ± 1.49	.50 (394)^(a)^
Self‐reported physical activity (IPAQ MET‐min/week)	3193.70 ± 2829.86	3186.46 ± 2964.91	3519.66 ± 2822.11	.51 (393.5)^(a)^
Disability indicators				
Self‐reported health				
Poor	29 (48)	8 (23)	21 (84)	**<.001** (21.832)^(b)^
Good	31 (52)	27 (77)	4 (16)	
Hand grip strength (kg)	27.39 ± 8.56	36.59 ± 39.86	25.07 ± 7.54	.**02** (279.5)^(a)^
One Leg Standing time (s)	30.32 ± 22.77	38.83 ± 20.93	18.19 ± 20.72	**<.001** (192.5)^(a)^
ADL independence (Barthel Index score)	19.86 ± .35	19.97 ± .17	19.76 ± .44	.**01** (345)^(a)^
IADL independence (Lawton and Brody score)	22.70 ± 1.23	23 ± .00	21.96 ± 2.67	.**002** (332.5)^(a)^

*Note*: Data represented by the mean and standard deviation for ordinal variables, and frequencies for nominal variables. The *p*‐value reflects the comparison between older adults without (ND) and with disability (D) by (a) Mann–Whitney *U* test, (b) *χ* ^2^ test, (c) Independent samples *t*‐test. Significance values are provided in bold.

Older adults with functional disability exhibited significantly higher peak AP CoM acceleration compared to their non‐disabled counterparts (Table 2).

**TABLE 2:** Task performance global characterisation of the sample.

	OA (*n* = 60)	ND (*n* = 35)	D (*n* = 25)	*p*‐Value (test value)
Sit‐ST time	2.57 ± .69	2.59 ± .74	2.55 ± .62	.94 (432.50)
Flexion time	.7 ± .17	.72 ± .16	.66 ± .18	.24 (359.00)
Momentum transfer time	.52 ± .14	.52 ± .15	1.34 ± .13	.75 (416.50)
Extension time	1.19 ± .58	1.19 ± .60	1.18 ± .56	.95 (433.50)
Stabilisation time	.16 ± .06	.15 ± .05	.18 ± .06	.04 (297.00)
Stand‐TS time	2.75 ± .73	2.88 ± .72	2.56 ± .70	.12 (334.00)
Initiation time	1.06 ± .50	1.11 ± .53	1.00 ± .46	.51 (394.00)
Flexion time	.30 ± .13	.32 ± .14	.27 ± .11	.32 (370.50)
Momentum transfer time	.17 ± .08	.17 ± .07	.18 ± .09	.30 (369.00)
Extension time	1.21 ± .35	1.29 ± .30	1.11 ± .38	.09 (323.50)
Peak AP CoM acceleration Sit‐TS	1.25 ± .48	1.10 ± .33	1.47 ± .57	.02 (276.00)
Peak AP CoM deceleration Stand‐TS	−.91 ± .71	−1.01 ± .89	−.77 ± .27	.39 (380.50)
AP postural sway				
Sit‐TS	.47 ± .15	.48 ± .17	.45 ± .11	.61 (403.00)
Stand‐TS	.36 ± .11	.36 ± .13	.35 ± .10	.93 (432.00)
ML postural sway				
Sit‐TS	.10 ± .03	.11 ± .04	.09 ± .02	.09 (324.00)
Stand‐TS	.12 ± .04	.12 ± .04	.12 ± .04	.84 (424.00)

*Note*: Data is represented by the mean and standard deviation. Overall Sit‐TS and Stand‐TS competition time and respective phases, peak centre of mass (CoM) acceleration for Sit‐TS and peak CoM deceleration for Stand‐TS, antero‐posterior (AP) and mediolateral (ML) postural sway are described. The *p*‐value reflects the comparison between older adults without (ND) and with disability (D) by Mann–Whitney *U* test.

The results of preliminary PCMs across the different phases of Sit‐TS and Stand‐TS are described in Tables S1–S8 of the Appendix S1.

**FIGURE 2:**
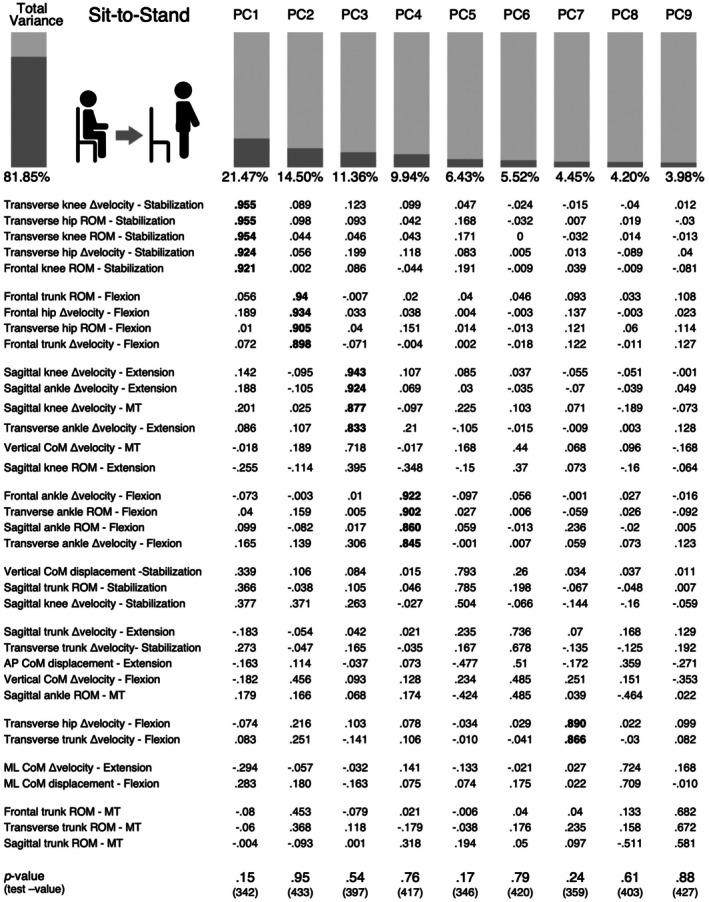
Principal component model of Sit‐to‐Stand task. Loadings above .8 are in bold and the *p*‐values correspond to the differences between older adults with and without disability according to the Mann–Whitney *U* test. MT, Momentum transfer phase; ROM, Range of motion; Δ velocity, range of angular velocity.

For the Sit‐TS phases, the four PCMs yielded KMO values ranging from .545 to .842, confirming the suitability of the study sample for dimensionality reduction. Bartlett's test of Sphericity was also significant (*p* < .001). The PCMs explained between 76% and 86% of the data variance. A threshold of .8 was adopted a priori to identify parameters with high loading in each PCM. Applying this criterion, 34 kinematic parameters were selected to construct the Sit‐TS principal component model (Figure 2). This model explained 82% of the total variance, with sample adequacy confirmed by a KMO value of .597 and a significant Bartlett's test of Sphericity (*p* < .001). None of the PC scores revealed significant differences between older adults with and without functional disability.

**FIGURE 3:**
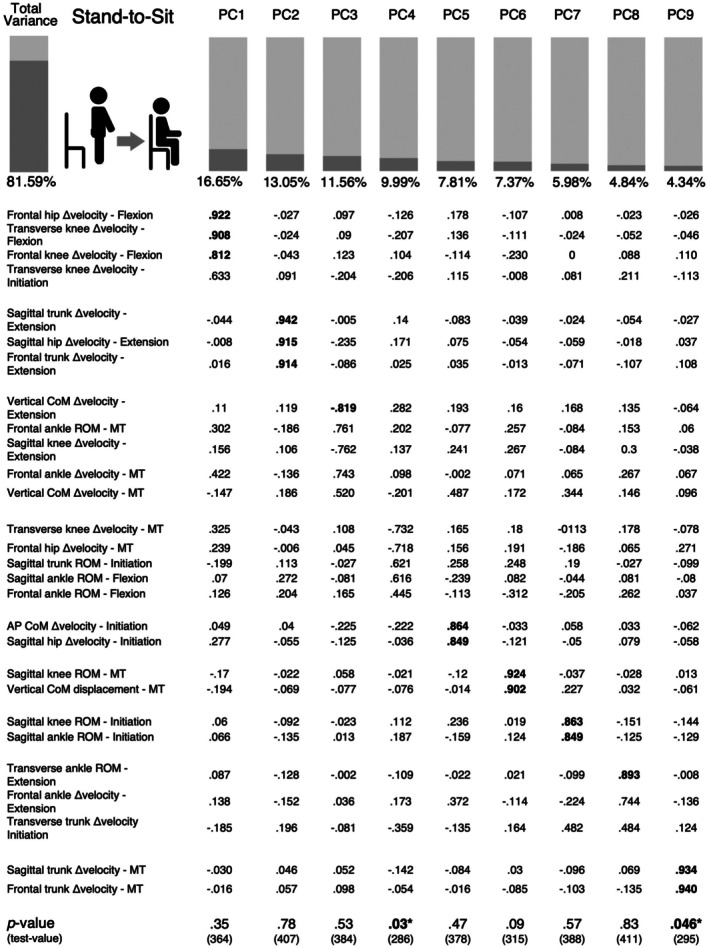
Principal component model of Stand‐to‐Sit task. Loadings above .8 are in bold and the *p*‐values correspond to the differences between older adults with and without disability according to the Mann–Whitney *U* test. MT, Momentum transfer phase; ROM, Range of motion; Δ velocity, range of angular velocity.

For the Stand‐TS task, outlier analysis identified one severe outlier across multiple kinematic parameters. All kinematic data for this participant were excluded from the model computation for the PCM across the four phases. The KMO values of these models ranged from .588 to .747, with Bartlett's test of Sphericity yielding *p* < .001. The total variance explained by each Stand‐TS phase ranged from 77% to 80%. Following the same procedure as the Sit‐TS PCM, 32 kinematic parameters with loadings higher than .8 in each PCM were gathered to construct the PCM for Stand‐TS. The final Stand‐TS PCM comprised 28 kinematic parameters explaining 82% of the total variance (Figure 3). Comparison of principal components scores between older adults with and without functional disability revealed significant group differences in the fourth and ninth components.

## 4 DISCUSSION

The aim of this cross‐sectional study was to examine the kinematics of Sit‐TS and Stand‐TS transitions among older adults and investigate differences in these domains between those with and without functional disability. Older adults with and without functional disability presented significant differences in all disability indicators assessed. Although BMI and handgrip strength are components of physical performance, we do not interpret the observed group differences as indicative of frailty status, which requires a more comprehensive multidimensional assessment. The comparative analysis of full task descriptive measures revealed significant group differences in peak AP CoM acceleration during Sit‐TS, with older adults with functional disability exhibiting higher acceleration compared to their non‐disabled counterparts. This increased acceleration, also seen in comparisons with healthy young adults, reflects a compensatory strategy to propel the upper body forward over the base of support and initiate seat‐off. This adaptation appears to result from reduced lower extremity force capacity, leading to increased acceleration to compensate and facilitate body elevation.^26^ The strategy is more pronounced in older adults with functional disabilities.

Principal component models were computed for tridimensional lower limb joints and trunk ROM, angular velocity ranges, and the CoM displacement and velocity ranges across different phases of Sit‐TS and Stand‐TS tasks. These PCMs were then integrated to construct comprehensive models for each task. The Sit‐TS PCM explained 82% of the total variance, emphasising the importance of parameters during the stabilisation phase, particularly the role of transverse hip and knee control. The Sit‐TS task poses a significant challenge as it involves transitioning from a large and stable base of support to a narrow and unstable one in the standing position.^2^ Early studies have underscored the need to further investigate stabilisation following the attainment of an upright posture.^27^ A recent study suggested that the stabilisation phase of Sit‐TS is particularly challenging for functionally independent older adults compared to younger adults, as evidenced by analyses of centre of pressure displacement.^28^ This difficulty may be attributed to several age‐related factors, including diminished proprioceptive feedback, slower neuromuscular response times and reduced ankle joint flexibility.^29^, ^30^ Additionally, physiological phenomena such as orthostatic hypotension could transiently affect blood pressure regulation upon standing, further complicating balance and contributing to postural instability during this phase.^31^

The flexion phase also emerges as critical in older adults' performance of the Sit‐TS task, as highlighted in principal components two, four and seven, which collectively accounted for 29% of the total variance. Although this phase is often characterised by forward trunk transfer and maximum hip flexion,^32^ the PCA in this study emphasised the importance of frontal and transverse trunk and hip control, underscoring the role of core trunk stability and coordination throughout the transition. A recent systematic review reported increased trunk flexion in older adults compared to younger individuals and highlighted the need for comprehensive analyses of movements in the frontal and transverse planes.^2^ Joint functioning in these planes may be key to identifying age‐related differences in balance regulation during the Sit‐TS task.^2^ The importance of frontal hip control, possibly reflecting compensatory trunk stabilisation, might be associated with age‐related weakening of the hip abductors, which are known to influence both gait and stabilisation at seat‐off.^33^ The third PC, reflecting sagittal knee control during momentum transfer and extension and sagittal and transverse ankle velocity range at extension, reinforces age‐related compensations of knee and ankle musculature.^34^ Although anticipatory postural adjustments were not directly analysed, the demands of transitioning from potentially stabilising postures (e.g. knee hyperextension) may contribute to the observed increased activation during the extension phase. Literature reports higher activation of rectus femoris, hamstrings, gastrocnemius and soleus in older adults compared to younger adults, possibly compensating for reduced trunk flexion while standing up.^34^

The Sit‐TS task is a bipodal movement characterised by significant kinematic changes in the sagittal plane, which has traditionally been the primary focus of research.^2^, ^35^ However, the present study PCA revealed that transverse and frontal plane control of the trunk, hip and knee joints plays a significant role in the movement. Notably, the critical sagittal plane kinematics of the knee and ankle were retained in the third principal component (PC), indicating their contribution was not among the primary sources of variability. This finding suggests that the parameters contributing most to the components explaining the majority of variance are not necessarily the most relevant for achieving the task goal. In other words, individual variability in this task appears to be more associated with less critical parameters. This observation aligns with Uncontrolled Manifold (UCM) theory, which posits that the central nervous system organises variability to ensure stability in task‐relevant dimensions while allowing variability in other, less goal‐related dimensions.^36^, ^37^ According to the UCM theory, the central nervous system utilises a manifold of solutions to achieve a motor task, allowing for flexibility and robustness in movement.^36^, ^37^ The UCM theory has previously been applied to the study of the Sit‐TS task, highlighting that the position of the CoM in the sagittal plane is tightly controlled, while the horizontal head position and the position of the hand were controlled less stably.^38^, ^39^ The present PCA emphasises the key sagittal plane parameters required for the task goal. Meanwhile, similar to the head and hand positions, the transverse and frontal plane parameters, which contribute less to the task goal, are permitted greater variability. This reflects a prioritisation of task‐relevant stability over strict control of all movement dimensions.

The Stand‐TS PCM presented similar total variance explained as the Sit‐TS model, specifically 82%, with the first component gathering parameters of the flexion phase. The first PC, which accounted for the largest variance, included hip and knee frontal and transverse angular velocity during this phase. The second component consisted of sagittal hip and trunk angular velocity, while the third component included the vertical CoM angular velocity range, all measured during the extension phase. In contrast with the Sit‐TS task, the PCA for its reverse function revealed that task‐relevant kinematics were among those explaining the largest variance, primarily in the second and third components. Additionally, the fifth component (AP CoM angular velocity range and sagittal hip angular velocity range at initiation) and seventh component (sagittal knee and ankle ROM) also included task‐relevant kinematics.

As for the flexion phase, the majority of studies highlight the decrease in trunk flexion of older adults compared to younger ones.^40^, ^41^ Decreased trunk flexion, combined with impaired eccentric control of knee extensors, minimises forward body displacement during sitting and has been linked to Stand‐TS instability in ageing. This is considered an adaptive mechanism to reduce the risk of anterior disequilibrium.^40^, ^41^ Other biomechanical factors may also contribute to this movement pattern. Notably, reduced flexibility of the hamstring muscles, which can limit anterior pelvic tilt and trunk flexion, may mechanically constrain the forward bending required for controlled descent. Age‐related increases in passive stiffness of the posterior chain could therefore restrict trunk mobility and alter sitting strategies.^42^ However, in this PCA, sagittal trunk ROM during the flexion phase did not load higher than .8 on the flexion phase PCM and thus was excluded from the Stand‐TS PCM. Instead, sagittal and frontal trunk angular velocity range at momentum transfer were included in the model under the ninth PC. This finding suggests that beyond the well‐documented reduction in sagittal trunk ROM,^40^ trunk motion in the frontal plane warrants further investigation. In older adults with functional disability, these parameters differ significantly compared with older adults without disability. Studies on stroke patients often report increased lateral trunk flexion toward the non‐paretic side.^43^ However, the role and significance of frontal plane trunk motion in older adults with functional disabilities remain to be thoroughly explored.

Comparisons of PC scores between disability groups suggest that the Stand‐TS task is more discriminative in distinguishing older adults with and without functional disabilities, as two PCs showed significant differences. This greater discriminative power may relate not only to the task's general reliance on eccentric control but also to the specific kinematic features captured by the fourth and ninth principal components. The fourth PC likely reflects complex multi‐planar control challenges during descent. Older adults with disabilities may have difficulty modulating these subtle rotations, especially given that transverse and frontal plane control are more vulnerable to age‐related neuromuscular decline^44^ that may increase instability and fall risk.^40^ Similarly, PC9 may reflect trunk coordination. The need to stabilise the trunk while lowering the CoM requires continuous feedback and feedforward control, functions that are often compromised in populations with disabilities due to diminished proprioception, delayed muscle activation or reduced core strength.^45^, ^46^ These findings align with recent literature, which recommends incorporating Stand‐TS training into comprehensive balance interventions,^40^ despite the Sit‐TS task being more commonly assessed and rehabilitated.^47^ Furthermore, the results of the PCMs for each task support the importance of evaluating the Stand‐TS task in older adults. While in the Sit‐TS task, the principal components include parameters that are less directly relevant to the task goal, in the Stand‐TS task, the parameters explaining the greatest variance are more critical to achieving the task. Prior work has shown that younger adults typically exhibit greater trunk flexion, smoother momentum transfer, and more stable deceleration during Stand‐TS transitions,^40^ highlighting the age‐related adaptations observed in this older sample.

This study should be interpreted while acknowledging its limitations, particularly the sample size, which may affect the generalisability of PCA results. To address this, rigorous measures were implemented to ensure sampling adequacy for dimensionality reduction across all models employed. Data suitability was confirmed (KMO >.5), as recommended by Hair et al (1998). Additionally, it is important to note that the tasks were performed without hand support and with foot placement determined by the participant. While standardisation was ensured for the assessed sample, it is well‐established that the ability to perform a Sit‐TS movement is influenced by these and other factors, such as chair seat height and the use of armrests.^8^ Particularly, the fixed chair height reflects real‐life conditions, but it does not account for individual differences in participant height and leg length. For shorter individuals, the relative effort required to stand from a fixed‐height chair may be greater, potentially influencing performance metrics. This variation could have contributed to within‐group variability and should be considered when interpreting results. Additionally, participants were instructed to cross their arms across the chest during the tasks to minimise variability in upper‐limb movement and ensure clearer kinematic analysis of trunk and lower‐limb motion. While this standardisation is common in biomechanical studies and helps isolate lower‐body performance, it restricts natural compensatory strategies, such as using arm swing for momentum.^34^

Furthermore, this study incorporated factors not commonly considered in previous research on age‐related impacts, such as the range of activity levels.^2^ Notably, this study confirmed that there were no differences in self‐reported physical activity between the two groups of older adults, ensuring that differences observed are not confounded by variations in activity levels. Nevertheless, heterogeneity may be present within the disability group. Participants classified as having a disability presented with two or more indicators, meaning that functional impairment could vary considerably between individuals. This variability may have influenced the way tasks were performed, with those presenting a higher number of indicators potentially experiencing more pronounced functional limitations. Future studies could consider stratifying participants based on the number or type of disability indicators to better understand how different levels of impairment affect performance.

The findings of the present study, particularly the discriminative value of Stand‐TS in identifying functional disability, offer relevant implications for clinical and rehabilitation contexts. The observed differences in transverse and frontal plane control (PC4 and PC9) suggest that these movement domains may serve as sensitive markers of decline. Incorporating Stand‐TS assessments into clinical evaluations may enhance early identification of individuals at risk for mobility loss or falls. Moreover, rehabilitation programs could benefit from targeted training focused on eccentric control and multi‐planar stability during descent, which are often underemphasised in standard protocols. This aligns with emerging evidence supporting task‐specific, phase‐targeted interventions in older adults.^47^

## 5 CONCLUSIONS

The kinematics of Sit‐TS and Stand‐TS transitions in older adults are explained through PCMs, each consisting of nine PCs, accounting for over eighty percent of the variance. The Stand‐TS emerges as the most discriminative task in distinguishing older adults with and without functional disability.

## FUNDING INFORMATION

This work was supported by the Fundação para a Ciência e Tecnologia (FCT), Portuguese Ministry of Education, Science and Innovation, NORTE 2020, the European Social Fund of the European Union, grant number 2020.05356.BD (DOI 10.54499/2020.05356.BD) and through R&D Units funding (UIDB/05210/2020), Fundação para a Ciência e Tecnologia (FCT), Portugal and the European Union.

## CONFLICT OF INTEREST STATEMENT

No conflicts of interest declared.

## Supporting information


Appendix S1

## Data Availability

The dataset generated and/or analysed during this study is not publicly available due to the confidentiality of the data.
