# Supplementary material for: Sit‐to‐stand and stand‐to‐sit kinematics in older adults with and without functional disability: A principal component analysis
Source: Australas J Ageing. 2025 Sep 2;44(3):e70089. doi: 10.1111/ajag.70089 (PMC12404020; doi:10.1111/ajag.70089)
Supplement: Supplementary file 1 — Appendix S1 [file AJAG-44-0-s001.docx]

**Supplementary material**

Sit-To-Stand (Sit-TS) and Stand-To-Sit (Stand-TS) phases

The Sit-TS and Stand-TS tasks were divided into four phases each, according previous literature on older adults movement analysis (Lin and Lee 2022) (**Figure 1**). The Sit-TS was divided into flexion, momentum transfer, extension and stabilisation (Lin and Lee 2022). The flexion or initiation phase started from the first deflection of vertical ground reaction force (GRF) following the start of the acquisition time (Piano et al. 2020) and ended at seat-off event, corresponding to the maximum positive anteroposterior GRF following initiation (Buckley et al. 2009). The momentum transfer phase started at seat-off and ended at maximum peak vertical centre of mass (CoM) velocity (Buckley et al. 2009). The third phase, extension, begins at maximum peak vertical CoM velocity and ended at extension, that was defined as the instant when the sagittal knee angle reaches the minimum values, as the maximum point of knee extension (Alcock et al. 2015). The stabilisation phase started at the end of the previous phase and ended at a stabilisation event. Although the stabilisation point has been defined by visual inspection in the literature (Piano et al. 2020), to standardise to all participants, in this study it was defined as the instant the vertical force reached the average value calculated in the first 50 frames after the extension (standing baseline).

The Stand-TS task was divided into initiation, flexion, momentum transfer and extension (Lin and Lee 2022). The start of sitting down was defined by the beginning of knee flexion, which was defined as the instant where sagittal knee angle reached a value higher than the average value of the standing baseline plus two times the standard deviation. The initiation phase started from the previous event and ended at minimum peak vertical CoM velocity. This event initiated the flexion phase, that ended at the maximum peak vertical GRF (Lin and Lee 2022). The momentum transfer phase started at the end of the previous phase and ended at seat-on event, corresponding to minimum anteroposterior GRF. The extension phase began at seat-on and ended at the instant where the vertical GRF assumed a value lower than the average value obtained during the first 50 frames after seat-on plus two times the standard deviation.

**Figure. S1**: Sit-to-Stand and Stand-to-Sit phases and respective events defined according to vertical and anteroposterior Ground Reaction Force (GRF), sagittal knee angle and vertical centre of mass velocity.


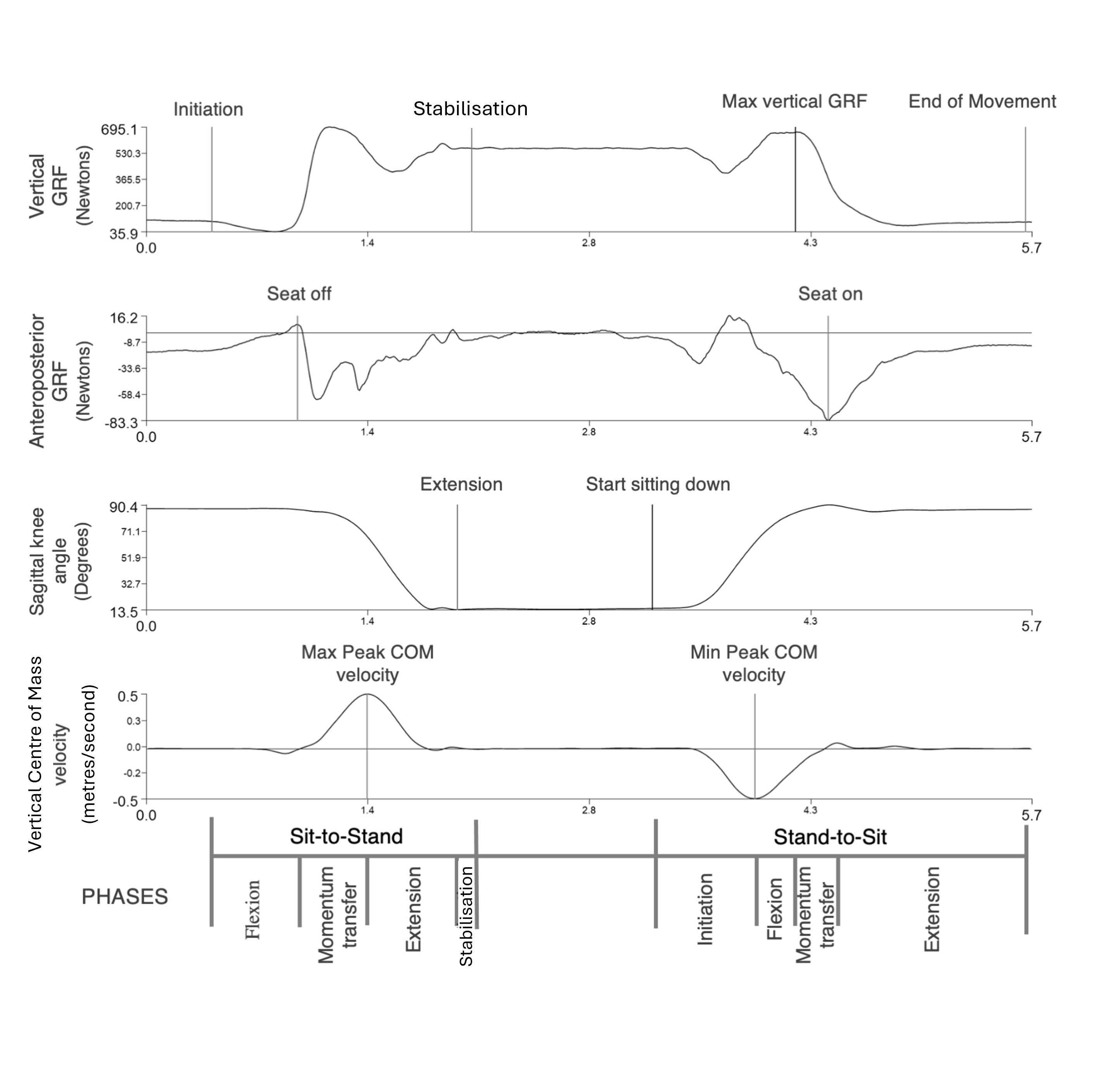


**Principal Component Models of Sit-TS phases**

**Table S1:** Principal component model of trunk, hip, knee and ankle range of motion (ROM), joint velocity range in sagittal, frontal and transverse planes, and centre of mass (CoM) displacement and velocity range in anteroposterior (AP), mediolateral (ML) and vertical directions during the Flexion phase of Sit-TS task. Kaiser-Meyer-Olkin value of 0.743 and Bartlett’s Test of Sphericity < 0.05. Parameters with loadings > 0.800 are in bold.

| **Flexion Phase Sit-TS** |  |  |  |  |  |  |
| --- | --- | --- | --- | --- | --- | --- |
| Principal Component | 1 | 2 | 3 | 4 | 5 | 6 |
| Explained variance (%) | 38 | 16 | 8 | 6 | 5 | 4 |
|  |  |  |  |  |  |  |
| **Sagittal ankle ROM** | **0.871** | -0.053 | 0.199 | 0.090 | -0.029 | 0.072 |
| **Frontal ankle joint velocity range** | **0.860** | -0.040 | -0.048 | 0.202 | 0.054 | 0.155 |
| **Transverse ankle joint velocity range** | **0.849** | 0.191 | 0.064 | 0.202 | -0.015 | -0.019 |
| **Transverse ankle ROM** | **0.832** | 0.117 | -0.096 | 0.120 | 0.066 | 0.231 |
| Transverse knee ROM | 0.734 | 0.078 | 0.110 | -0.013 | 0.310 | 0.059 |
| Frontal ankle ROM | 0.731 | -0.115 | -0.048 | 0.059 | 0.368 | 0.179 |
| Frontal knee joint velocity range | 0.713 | 0.207 | 0.366 | 0.076 | 0.107 | -0.226 |
| Sagittal ankle joint velocity range | 0.711 | -0.008 | 0.186 | 0.398 | 0.022 | -0.133 |
| Sagittal knee joint velocity range | 0.634 | 0.540 | 0.078 | 0.033 | -0.095 | 0.251 |
|  |  |  |  |  |  |  |
| **Frontal hip joint velocity range** | 0.025 | **0.931** | 0.182 | 0.094 | 0.092 | 0.096 |
| **Transverse hip ROM** | 0.141 | **0.904** | 0.133 | 0.061 | 0.200 | 0.137 |
| **Frontal trunk ROM** | -0.027 | **0.901** | 0.123 | -0.008 | 0.262 | 0.128 |
| **Frontal trunk joint velocity range** | -0.052 | **0.887** | 0.188 | 0.060 | 0.093 | 0.045 |
| Sagittal trunk joint velocity range | -0.023 | 0.514 | 0.283 | 0.462 | 0.114 | -0.123 |
| Frontal knee ROM | 0.288 | 0.567 | 0.122 | 0.265 | -0.021 | 0.330 |
| Transverse knee joint velocity range | 0.333 | 0.503 | 0.378 | 0.169 | -0.167 | 0.056 |
|  |  |  |  |  |  |  |
| **Transverse trunk joint velocity range** | 0.055 | 0.205 | **0.887** | 0.160 | -0.004 | 0.072 |
| **Transverse hip joint velocity range** | 0.122 | 0.134 | **0.866** | 0.075 | 0.207 | -0.006 |
| Transverse trunk ROM | 0.002 | 0.292 | 0.780 | 0.134 | 0.211 | 0.189 |
| Frontal hip ROM | 0.231 | 0.254 | 0.555 | 0.07 | 0.471 | 0.038 |
|  |  |  |  |  |  |  |
| Sagittal hip ROM | 0.401 | 0.11 | 0.118 | 0.771 | 0.084 | 0.150 |
| Ap CoM velocity range | 0.364 | 0.103 | 0.130 | 0.739 | 0.387 | -0.023 |
| Sagittal hip joint velocity range | 0.037 | 0.592 | 0.169 | 0.640 | 0.294 | 0.005 |
| AP CoM displacement | 0.397 | 0.047 | 0.227 | 0.594 | 0.307 | 0.403 |
|  |  |  |  |  |  |  |
| **Vertical CoM velocity range** | 0.088 | 0.240 | 0.098 | 0.313 | **0.812** | 0.033 |
| Vertical CoM displacement | 0.210 | 0.014 | 0.287 | 0.332 | 0.708 | 0.145 |
| Sagittal trunk ROM | 0.082 | 0.467 | 0.223 | 0.009 | 0.668 | 0.090 |
|  |  |  |  |  |  |  |
| **ML CoM displacement** | 0.006 | 0.113 | 0.046 | 0.043 | 0.069 | **0.860** |
| Sagittal knee ROM | 0.345 | 0.364 | 0.140 | 0.004 | 0.064 | 0.568 |
| ML CoM velocity range | 0.169 | 0.245 | 0.500 | 0.147 | 0.194 | 0.502 |

**Table S2:** Principal component model of trunk, hip, knee and ankle range of motion (ROM), joint velocity range in sagittal, frontal and transverse planes, and centre of mass (CoM) displacement and velocity range in anteroposterior (AP), mediolateral (ML) and vertical directions during the Momentum transfer phase of Sit-TS task. Kaiser-Meyer-Olkin value of 0.545 and Bartlett’s Test of Sphericity < 0.05. Parameters with loadings > 0.800 are in bold.

| **Momentum transfer phase Sit-TS** | | | | | | | | | |
| --- | --- | --- | --- | --- | --- | --- | --- | --- | --- |
| Principal Component | 1 | 2 | 3 | 4 | 5 | 6 | 7 | 8 | 9 |
| Explained variance (%) | 23 | 13 | 10 | 9 | 6 | 5 | 4 | 4 | 4 |
|  |  |  |  |  |  |  |  |  |  |
| **Sagittal knee joint velocity range** | **0.924** | 0.003 | 0.151 | -0.118 | -0.029 | -0.008 | -0.015 | -0.055 | 0.080 |
| **Vertical CoM velocity** | **0.864** | 0.175 | -0.051 | 0.099 | -0.112 | 0.145 | -0.066 | 0.094 | -0.156 |
| Sagittal hip joint velocity range | 0.769 | -0.121 | 0.057 | 0.401 | 0.298 | 0.008 | -0.072 | 0.076 | -0.224 |
| Vertical CoM displacement | 0.703 | 0.203 | 0.109 | 0.100 | -0.233 | 0.135 | 0.450 | -0.008 | 0.258 |
| Sagittal ankle joint velocity range | 0.682 | -0.102 | 0.285 | -0.007 | 0.022 | -0.013 | -0.112 | 0.330 | 0.348 |
| Sagittal hip ROM | 0.625 | -0.149 | 0.210 | 0.352 | 0.254 | -0.038 | 0.442 | -0.145 | 0.016 |
|  |  |  |  |  |  |  |  |  |  |
| **Frontal trunk ROM** | -0.157 | **0.844** | 0.115 | -0.004 | -0.017 | -0.003 | -0.064 | -0.015 | 0.109 |
| **Transverse trunk ROM** | 0.079 | **0.824** | -0.042 | 0.146 | -0.003 | -0.015 | 0.019 | -0.002 | 0.033 |
| Transverse trunk joint velocity range | 0.105 | 0.629 | 0.092 | 0.024 | 0.042 | 0.118 | 0.170 | 0.159 | 0.174 |
| Frontal trunk joint velocity range | -0.167 | 0.627 | 0.072 | -0.070 | 0.200 | 0.398 | -0.149 | 0.197 | -0.163 |
| Frontal hip ROM | 0.187 | 0.577 | 0.080 | 0.132 | -0.253 | 0.483 | 0.163 | 0.309 | -0.138 |
| Transverse hip joint velocity range | 0.324 | 0.485 | 0.181 | 0.424 | 0.028 | 0.313 | -0.054 | 0.171 | 0.027 |
| Frontal ankle ROM | 0.275 | 0.406 | -0.146 | 0.533 | -0.054 | -0.049 | 0.205 | 0.393 | 0.055 |
|  |  |  |  |  |  |  |  |  |  |
| Transverse knee joint velocity range | 0.124 | -0.009 | 0.787 | 0.225 | 0.187 | -0.015 | -0.229 | 0.138 | 0.056 |
| Transverse hip ROM | 0.224 | 0.350 | 0.733 | 0.107 | 0.111 | -0.057 | 0.249 | -0.229 | -0.058 |
| Frontal knee ROM | -0.069 | -0.085 | 0.728 | -0.289 | -0.317 | 0.097 | 0.061 | 0.044 | 0.080 |
| Frontal hip joint velocity range | 0.100 | 0.301 | 0.691 | 0.387 | 0.274 | 0.139 | -0.055 | 0.041 | -0.075 |
| Frontal knee joint velocity range | 0.415 | -0.013 | 0.614 | 0.024 | 0.181 | 0.301 | -0.198 | -0.138 | 0.091 |
|  |  |  |  |  |  |  |  |  |  |
| **Sagittal ankle ROM** | 0.148 | -0.047 | 0.113 | **0.832** | 0.196 | -0.108 | -0.096 | 0.108 | 0.207 |
| AP CoM displacement | -0.006 | 0.226 | 0.178 | 0.788 | -0.316 | 0.064 | 0.168 | 0.146 | -0.067 |
|  |  |  |  |  |  |  |  |  |  |
| Transverse ankle ROM | -0.036 | 0.191 | 0.282 | 0.091 | 0.708 | 0.136 | 0.138 | 0.203 | 0.070 |
| Tranverse ankle joint velocity range | 0.357 | 0.057 | 0.341 | 0.127 | 0.659 | -0.102 | -0.222 | 0.126 | 0.080 |
| AP CoM velocity range | 0.207 | 0.165 | 0.228 | 0.227 | -0.626 | -0.050 | -0.216 | 0.231 | 0.073 |
|  |  |  |  |  |  |  |  |  |  |
| Transverse knee ROM | 0.176 | -0.059 | 0.226 | -0.083 | -0.010 | 0.753 | 0.086 | -0.247 | 0.187 |
| Sagittal trunk joint velocity range | -0.030 | 0.406 | -0.062 | -0.015 | 0.108 | 0.746 | 0.033 | 0.143 | 0.113 |
|  |  |  |  |  |  |  |  |  |  |
| ML CoM displacement | -0.060 | 0.028 | -0.163 | 0.023 | 0.136 | 0.023 | 0.755 | 0.121 | 0.065 |
| Sagittal knee angle | 0.308 | 0.036 | 0.211 | -0.464 | -0.257 | 0.235 | 0.523 | -0.150 | 0.259 |
| ML CoM velocity range | -0.094 | 0.085 | -0.003 | 0.117 | -0.054 | 0.057 | 0.452 | 0.626 | -0.227 |
|  |  |  |  |  |  |  |  |  |  |
| Frontal ankle joint velocity range | 0.161 | 0.216 | -0.004 | 0.228 | 0.144 | -0.054 | -0.063 | 0.772 | 0.100 |
|  |  |  |  |  |  |  |  |  |  |
| **Sagittal trunk ROM** | 0.013 | 0.194 | 0.031 | 0.100 | 0.043 | 0.186 | 0.096 | -0.023 | **0.859** |

**Table S3:** Principal component model of trunk, hip, knee and ankle range of motion (ROM), joint velocity range in sagittal, frontal and transverse planes, and centre of mass (CoM) displacement and velocity range in anteroposterior (AP), mediolateral (ML) and vertical directions during the Extension phase of Sit-TS task. Kaiser-Meyer-Olkin value of 0.586 and Bartlett’s Test of Sphericity < 0.05. Parameters with loadings > 0.800 are in bold.

| **Extension phase Sit-TS** |  |  |  |  |  |  |  |  |
| --- | --- | --- | --- | --- | --- | --- | --- | --- |
| Principal Component | 1 | 2 | 3 | 4 | 5 | 6 | 7 | 8 |
| Explained variance (%) | 22 | 14 | 10 | 9 | 7 | 6 | 5 | 4 |
|  |  |  |  |  |  |  |  |  |
| **Sagittal knee joint velocity range** | **0.925** | 0.132 | -0.079 | 0.069 | -0.179 | -0.15 | -0.084 | -0.082 |
| **Sagittal ankle joint velocity range** | **0.904** | 0.120 | -0.167 | -0.011 | -0.183 | -0.111 | -0.015 | -0.001 |
| **Transverse ankle joint velocity range** | **0.828** | 0.010 | -0.043 | -0.050 | -0.222 | 0.070 | 0.199 | 0.041 |
| Sagittal hip joint velocity range | 0.779 | -0.126 | 0.416 | 0.120 | 0.046 | -0.035 | -0.085 | -0.166 |
| Vertical CoM velocity | 0.763 | 0.369 | 0.219 | 0.245 | 0.036 | -0.139 | 0.043 | -0.122 |
| Frontal ankle joint velocity | 0.718 | 0.062 | 0.270 | -0.083 | 0.015 | 0.283 | 0.180 | -0.255 |
| Transverse hip joint velocity range | 0.704 | -0.182 | 0.046 | 0.066 | 0.434 | -0.014 | 0.037 | 0.265 |
| Frontal knee joint velocity range | 0.654 | 0.122 | -0.142 | -0.001 | 0.059 | -0.032 | 0.166 | 0.514 |
| Frontal hip joint velocity range | 0.647 | -0.197 | -0.116 | 0.158 | 0.375 | 0.004 | 0.303 | 0.132 |
| Transverse knee joint velocity range | 0.529 | -0.260 | 0.011 | -0.149 | 0.467 | -0.116 | -0.209 | 0.395 |
|  |  |  |  |  |  |  |  |  |
| **Sagittal knee ROM** | 0.145 | **0.915** | 0.100 | 0.002 | -0.087 | -0.079 | 0.047 | 0.087 |
| Sagittal ankle ROM | 0.311 | 0.759 | -0.259 | -0.058 | -0.242 | -0.112 | 0.054 | 0.176 |
| Vertical CoM displacement | -0.209 | 0.725 | 0.289 | 0.215 | 0.244 | 0.170 | 0.093 | 0.066 |
| Sagittal trunk ROM | -0.275 | 0.555 | -0.081 | 0.456 | 0.231 | 0.251 | -0.208 | 0.049 |
|  |  |  |  |  |  |  |  |  |
| **Ap CoM displamencent** | 0.032 | -0.008 | **0.883** | 0.210 | 0.110 | 0.115 | 0.073 | 0.011 |
| AP CoM velocity range | 0.190 | -0.100 | 0.771 | 0.199 | -0.075 | 0.117 | -0.124 | -0.181 |
| Sagittal hip ROM | -0.119 | 0.314 | 0.753 | -0.121 | 0.174 | 0.142 | 0.184 | 0.153 |
|  |  |  |  |  |  |  |  |  |
| **Sagittal trunk joint velocity range** | 0.031 | 0.140 | 0.178 | **0.847** | -0.033 | -0.026 | -0.063 | -0.245 |
| Transverse trunk joint velocity range | 0.144 | 0.033 | 0.248 | 0.752 | -0.286 | -0.174 | 0.098 | -0.006 |
| Frontal trunk joint velocity range | -0.061 | -0.217 | -0.028 | 0.687 | 0.101 | 0.233 | 0.391 | 0.149 |
| Transverse trunk ROM | 0.193 | 0.217 | 0.025 | 0.583 | 0.035 | 0.435 | -0.046 | 0.281 |
|  |  |  |  |  |  |  |  |  |
| Transverse hip ROM | -0.016 | 0.034 | 0.045 | -0.024 | 0.764 | -0.057 | 0.358 | -0.010 |
| Transverse knee ROM | -0.117 | 0.018 | 0.114 | -0.083 | 0.747 | 0.014 | -0.125 | 0.151 |
|  |  |  |  |  |  |  |  |  |
| **ML CoM velocity range** | 0.025 | 0.000 | 0.174 | 0.086 | 0.090 | **0.855** | 0.014 | -0.194 |
| ML CoM displacement | -0.206 | -0.039 | 0.154 | -0.029 | -0.151 | 0.756 | 0.065 | 0.119 |
| Transverse ankle ROM | 0.168 | 0.302 | -0.294 | 0.340 | -0.017 | 0.361 | 0.178 | 0.224 |
|  |  |  |  |  |  |  |  |  |
| Frontal hip ROM | 0.219 | -0.033 | -0.101 | 0.252 | 0.129 | -0.071 | 0.735 | 0.002 |
| Frontal trunk ROM | -0.069 | 0.054 | 0.286 | -0.009 | -0.046 | 0.126 | 0.650 | 0.244 |
| Frontal ankle ROM | 0.212 | 0.322 | -0.056 | -0.149 | 0.038 | 0.099 | 0.514 | -0.309 |
|  |  |  |  |  |  |  |  |  |
| Frontal knee ROM | -0.074 | 0.245 | -0.015 | -0.017 | 0.191 | 0.010 | 0.074 | 0.759 |

**Table S4:** Principal component model of trunk, hip, knee and ankle range of motion (ROM), joint velocity range in sagittal, frontal and transverse planes, and centre of mass (CoM) displacement and velocity range in anteroposterior (AP), mediolateral (ML) and vertical directions during the Stabilisation phase of Sit-TS task. Kaiser-Meyer-Olkin value of 0.842 and Bartlett’s Test of Sphericity < 0.05. Parameters with loadings > 0.800 are in bold.

| **Stabilisation phase Sit-TS** |  |  |  |  |  |
| --- | --- | --- | --- | --- | --- |
| Principal Component | 1 | 2 | 3 | 4 | 5 |
| Explained variance (%) | 60 | 10 | 7 | 5 | 4 |
|  |  |  |  |  |  |
| **Transverse knee joint velocity range** | **0.925** | 0.102 | 0.229 | 0.138 | 0.167 |
| **Transverse hip ROM** | **0.921** | 0.238 | 0.157 | 0.095 | 0.189 |
| **Transverse knee ROM** | **0.901** | 0.245 | 0.168 | 0.074 | 0.218 |
| **Transverse hip joint velocity range** | **0.881** | 0.153 | 0.313 | 0.189 | 0.083 |
| **Frontal knee ROM** | **0.833** | 0.204 | 0.24 | 0.078 | 0.266 |
| Frontal trunk ROM | 0.668 | 0.284 | 0.397 | 0.312 | 0.154 |
|  |  |  |  |  |  |
| **Sagittal trunk ROM** | 0.222 | **0.878** | 0.149 | 0.190 | 0.113 |
| **Vertical CoM displacement** | 0.168 | **0.861** | 0.095 | 0.297 | 0.203 |
| Vertical CoM velocity | 0.236 | 0.776 | 0.455 | 0.181 | 0.224 |
| ML CoM velocity | 0.270 | 0.727 | 0.285 | 0.123 | 0.021 |
| Sagittal knee ROM | 0.148 | 0.684 | 0.432 | 0.010 | 0.466 |
| Sagittal hip ROM | 0.170 | 0.676 | 0.254 | 0.164 | 0.480 |
| Sagittal hip joint velocity range | 0.254 | 0.598 | 0.485 | 0.146 | 0.430 |
| ML CoM displacement | 0.059 | 0.595 | 0.326 | 0.105 | 0.043 |
|  |  |  |  |  |  |
| Sagittal ankle joint velocity range | 0.281 | 0.325 | 0.792 | 0.258 | 0.207 |
| Transverse ankle joint velocity range | 0.333 | 0.255 | 0.771 | 0.302 | 0.078 |
| AP CoM velocity range | 0.325 | 0.495 | 0.702 | 0.229 | 0.110 |
| Frontal ankle joint velocity range | 0.201 | 0.277 | 0.650 | 0.072 | 0.552 |
| Sagittal ankle ROM | 0.335 | 0.277 | 0.636 | 0.367 | 0.357 |
| Transverse ankle ROM | 0.359 | 0.490 | 0.613 | 0.108 | 0.148 |
| AP CoM displacement | 0.301 | 0.426 | 0.586 | 0.396 | 0.244 |
|  |  |  |  |  |  |
| **Transverse trunk joint velocity range** | 0.057 | 0.121 | 0.180 | **0.926** | 0.055 |
| Frontal trunk joint velocity range | 0.389 | 0.158 | 0.084 | 0.742 | 0.372 |
| Sagittal trunk joint velocity range | 0.210 | 0.438 | 0.365 | 0.727 | 0.018 |
| Transverse trunk ROM | 0.110 | 0.288 | 0.551 | 0.693 | 0.053 |
|  |  |  |  |  |  |
| **Sagittal knee joint velocity range** | 0.209 | 0.299 | 0.224 | 0.029 | **0.838** |
| Frontal knee joint velocity range | 0.515 | -0.017 | 0.164 | 0.247 | 0.751 |
| Frontal hip ROM | 0.512 | 0.441 | -0.008 | 0.153 | 0.540 |
| Frontal hip joint velocity range | 0.442 | 0.251 | 0.403 | 0.398 | 0.466 |
| Frontal ankle ROM | 0.339 | 0.384 | 0.498 | 0.212 | 0.427 |

**Principal Component Models of Stand-TS phases**

**Table S5:** Principal component model of trunk, hip, knee and ankle range of motion (ROM), joint velocity range in sagittal, frontal and transverse planes, and centre of mass (CoM) displacement and velocity range in anteroposterior (AP), mediolateral (ML) and vertical directions during the Initiation phase of Stand-TS task. Kaiser-Meyer-Olkin value of 0.588 and Bartlett’s Test of Sphericity < 0.05. Parameters with loadings > 0.800 are in bold.

| **Initiation phase Stand-TS** | | | | | | | | | | |
| --- | --- | --- | --- | --- | --- | --- | --- | --- | --- | --- |
| Principal component | 1 | 2 | 3 | 4 | 5 | 6 | 7 | 8 | 9 | 10 |
| Variance explained (%) | 24 | 11 | 9 | 8 | 6 | 6 | 5 | 4 | 4 | 3 |
|  |  |  |  |  |  |  |  |  |  |  |
| **AP CoM velocity** | **0.879** | 0.104 | -0.055 | 0.052 | 0.085 | -0.063 | -0.037 | 0.156 | -0.006 | 0.081 |
| **Sagittal hip velocity range** | **0.871** | 0.196 | -0.109 | 0.066 | -0.052 | 0.265 | -0.013 | 0.000 | -0.017 | 0.028 |
| Vertical CoM velocity | 0.774 | 0.200 | 0.228 | 0.070 | 0.084 | 0.340 | 0.190 | -0.071 | -0.112 | 0.138 |
| Sagittal knee velocity range | 0.669 | 0.175 | 0.293 | 0.054 | -0.075 | 0.553 | 0.066 | 0.004 | 0.007 | -0.083 |
| AP CoM displacement | 0.597 | 0.073 | -0.015 | 0.250 | 0.038 | -0.079 | 0.221 | 0.423 | 0.078 | 0.042 |
| Frontal trunk ROM | 0.459 | -0.151 | 0.013 | 0.332 | 0.344 | 0.043 | 0.017 | -0.091 | 0.229 | -0.445 |
|  |  |  |  |  |  |  |  |  |  |  |
| **Transverse knee velocity range** | 0.138 | **0.912** | -0.010 | 0.022 | 0.022 | 0.175 | -0.115 | -0.119 | -0.135 | -0.076 |
| **Transverse hip velocity range** | 0.100 | **0.863** | 0.059 | 0.010 | 0.043 | 0.069 | 0.017 | -0.004 | 0.007 | 0.177 |
| Frontal hip velocity range | 0.344 | 0.734 | 0.001 | 0.032 | -0.023 | 0.286 | -0.049 | -0.086 | -0.007 | 0.076 |
| Transverse knee ROM | -0.036 | 0.639 | -0.026 | 0.171 | 0.002 | -0.115 | -0.033 | 0.382 | 0.043 | -0.459 |
| Transverse hip ROM | -0.062 | 0.604 | -0.037 | 0.276 | -0.036 | -0.158 | -0.099 | 0.185 | 0.449 | -0.047 |
| Frontal knee velocity range | 0.382 | 0.571 | 0.393 | -0.193 | 0.103 | 0.222 | 0.084 | 0.028 | 0.208 | 0.106 |
|  |  |  |  |  |  |  |  |  |  |  |
| **Sagittal ankle ROM** | -0.141 | 0.026 | **0.907** | 0.016 | 0.063 | 0.178 | 0.033 | 0.044 | 0.115 | 0.055 |
| **Sagittal knee ROM** | 0.243 | 0.026 | **0.860** | 0.186 | 0.071 | 0.026 | 0.164 | 0.186 | -0.058 | -0.009 |
| Frontal knee ROM | 0.047 | 0.433 | 0.444 | -0.149 | 0.049 | -0.281 | 0.326 | -0.071 | 0.412 | 0.026 |
|  |  |  |  |  |  |  |  |  |  |  |
| **ML CoM velocity** | 0.142 | 0.120 | 0.052 | **0.825** | 0.186 | 0.216 | 0.159 | -0.184 | 0.068 | 0.087 |
| ML CoM displacement | 0.103 | -0.003 | 0.082 | 0.784 | -0.011 | 0.096 | 0.067 | 0.009 | 0.222 | -0.069 |
| Sagittal hip ROM | 0.537 | 0.128 | 0.213 | 0.539 | 0.034 | -0.193 | 0.138 | 0.318 | -0.252 | 0.089 |
|  |  |  |  |  |  |  |  |  |  |  |
| **Transverse trunk velocity range** | -0.064 | 0.006 | 0.132 | 0.072 | **0.853** | -0.015 | -0.128 | 0.024 | -0.140 | 0.121 |
| Transverse trunk ROM | 0.028 | 0.072 | 0.112 | -0.001 | 0.757 | 0.047 | 0.304 | -0.160 | 0.004 | -0.212 |
| Frontal trunk velocity range | 0.113 | 0.017 | -0.042 | 0.070 | 0.751 | 0.043 | -0.020 | 0.222 | 0.299 | -0.034 |
|  |  |  |  |  |  |  |  |  |  |  |
| Sagittal ankle velocity range | 0.159 | 0.294 | 0.257 | 0.156 | 0.043 | 0.700 | 0.013 | 0.068 | 0.071 | -0.009 |
| Frontal ankle velocity range | 0.317 | 0.006 | -0.048 | 0.022 | 0.124 | 0.576 | 0.301 | 0.492 | -0.023 | 0.088 |
| Transverse ankle velocity range | 0.107 | 0.168 | -0.227 | 0.385 | 0.092 | 0.564 | -0.343 | 0.115 | 0.271 | 0.184 |
|  |  |  |  |  |  |  |  |  |  |  |
| **Sagittal trunk ROM** | 0.098 | -0.140 | 0.223 | 0.163 | -0.004 | 0.035 | **0.840** | 0.015 | 0.125 | -0.101 |
| Vertical CoM displacement | 0.003 | 0.023 | 0.503 | 0.383 | 0.190 | -0.160 | 0.512 | 0.203 | -0.274 | 0.227 |
| Sagittal trunk velocity range | 0.330 | -0.028 | -0.325 | 0.158 | 0.447 | 0.231 | 0.489 | 0.057 | 0.049 | 0.261 |
|  |  |  |  |  |  |  |  |  |  |  |
| **Frontal ankle ROM** | 0.131 | -0.049 | 0.244 | -0.143 | 0.038 | 0.167 | -0.035 | **0.826** | 0.047 | 0.042 |
|  |  |  |  |  |  |  |  |  |  |  |
| Transverse ankle ROM | -0.061 | -0.006 | 0.051 | 0.199 | 0.087 | 0.127 | 0.081 | 0.019 | 0.790 | 0.108 |
|  |  |  |  |  |  |  |  |  |  |  |
| Frontal hip ROM | 0.371 | 0.095 | 0.131 | 0.147 | -0.028 | 0.067 | -0.058 | 0.109 | 0.308 | 0.653 |

**Table S6:** Principal component model of trunk, hip, knee and ankle range of motion (ROM), joint velocity range in sagittal, frontal and transverse planes, and centre of mass (CoM) displacement and velocity range in anteroposterior (AP), mediolateral (ML) and vertical directions during the Flexion phase of Stand-TS task. Kaiser-Meyer-Olkin value of 0.747 and Bartlett’s Test of Sphericity < 0.05. Parameters with loadings > 0.800 are in bold.

| **Flexion phase Stand-TS** |  |  |  |  |  |  |  |  |
| --- | --- | --- | --- | --- | --- | --- | --- | --- |
| Principal component | 1 | 2 | 3 | 4 | 5 | 6 | 7 | 8 |
| Variance explained (%) | 39 | 12 | 7 | 5 | 5 | 5 | 4 | 3 |
|  |  |  |  |  |  |  |  |  |
| **Transverse knee velocity range** | **0.903** | 0.042 | -0.002 | -0.143 | 0.032 | -0.090 | 0.251 | 0.037 |
| **Frontal hip velocity range** | **0.881** | 0.156 | -0.011 | -0.118 | 0.132 | -0.046 | 0.193 | 0.071 |
| **Frontal knee velocity range** | **0.846** | -0.010 | 0.250 | 0.064 | 0.210 | 0.082 | -0.068 | 0.095 |
| Transverse hip ROM | 0.778 | 0.088 | 0.005 | 0.246 | 0.032 | 0.251 | 0.226 | 0.006 |
| Transverse hip velocity range | 0.651 | 0.209 | 0.233 | 0.199 | 0.201 | 0.330 | -0.009 | -0.008 |
| Transverse ankle velocity range | 0.543 | 0.088 | 0.268 | 0.214 | 0.347 | 0.052 | -0.299 | 0.253 |
|  |  |  |  |  |  |  |  |  |
| Sagittal trunk velocity range | -0.033 | 0.775 | 0.229 | 0.046 | 0.157 | 0.210 | -0.177 | 0.060 |
| Frontal trunk velocity range | 0.009 | 0.743 | -0.002 | 0.156 | 0.088 | 0.128 | -0.175 | 0.256 |
| Vertical CoM velocity | 0.323 | 0.669 | 0.145 | 0.096 | 0.424 | 0.133 | 0.261 | 0.171 |
| Sagittal hip velocity range | 0.390 | 0.643 | 0.298 | 0.078 | 0.177 | -0.070 | 0.265 | 0.071 |
| Transverse trunk velocity range | 0.027 | 0.556 | 0.062 | 0.302 | -0.008 | 0.356 | -0.045 | 0.045 |
| Vertical CoM displacement | 0.298 | 0.467 | 0.379 | 0.431 | 0.194 | 0.253 | 0.393 | 0.203 |
|  |  |  |  |  |  |  |  |  |
| ML CoM displacement | 0.073 | 0.075 | 0.792 | 0.043 | 0.071 | 0.145 | -0.016 | 0.141 |
| ML CoM velocity | 0.094 | 0.114 | 0.724 | 0.097 | 0.250 | 0.176 | 0.167 | 0.314 |
| Transverse trunk ROM | 0.038 | 0.486 | 0.609 | 0.292 | -0.09 | 0.289 | 0.149 | -0.110 |
| Sagittal hip ROM | 0.351 | 0.466 | 0.591 | 0.244 | 0.062 | -0.094 | 0.268 | 0.112 |
| AP CoM displacement | 0.250 | 0.480 | 0.554 | -0.023 | 0.120 | 0.005 | 0.385 | 0.262 |
|  |  |  |  |  |  |  |  |  |
| **Sagittal ankle ROM** | -0.080 | 0.154 | 0.055 | **0.886** | 0.181 | -0.012 | 0.125 | 0.231 |
| Sagittal knee ROM | 0.196 | 0.390 | 0.189 | 0.725 | 0.172 | 0.164 | 0.346 | 0.093 |
| Transverse ankle ROM | 0.138 | 0.14 | 0.574 | 0.601 | 0.140 | 0.253 | -0.146 | -0.005 |
|  |  |  |  |  |  |  |  |  |
| **Sagittal ankle velocity range** | 0.271 | 0.118 | 0.012 | 0.240 | **0.853** | -0.034 | -0.038 | 0.148 |
| Sagittal knee velocity range | 0.376 | 0.325 | 0.094 | 0.143 | 0.778 | -0.017 | -0.020 | -0.045 |
| AP CoM velocity | -0.074 | 0.051 | 0.428 | -0.018 | 0.648 | 0.216 | 0.267 | 0.141 |
|  |  |  |  |  |  |  |  |  |
| Frontal trunk ROM | -0.037 | 0.123 | -0.011 | 0.029 | -0.077 | 0.768 | 0.066 | 0.354 |
| Frontal hip ROM | 0.125 | 0.228 | 0.345 | 0.142 | 0.073 | 0.720 | 0.111 | 0.025 |
| Sagittal trunk ROM | 0.300 | 0.206 | 0.319 | 0.015 | 0.195 | 0.603 | 0.176 | 0.114 |
|  |  |  |  |  |  |  |  |  |
| Frontal knee ROM | 0.442 | -0.212 | 0.06 | 0.134 | 0.025 | 0.117 | 0.674 | 0.123 |
| Transverse knee ROM | 0.170 | 0.023 | 0.256 | 0.244 | 0.047 | 0.249 | 0.569 | -0.241 |
|  |  |  |  |  |  |  |  |  |
| **Frontal ankle ROM** | 0.051 | 0.229 | 0.282 | 0.174 | 0.045 | 0.241 | 0.034 | **0.800** |
| Frontal ankle velocity range | 0.260 | 0.229 | 0.216 | 0.168 | 0.238 | 0.215 | -0.063 | 0.726 |

**Table S7:** Principal component model of trunk, hip, knee and ankle range of motion (ROM), joint velocity range in sagittal, frontal and transverse planes, and centre of mass (CoM) displacement and velocity range in anteroposterior (AP), mediolateral (ML) and vertical directions during the Momentum transfer phase of Stand-TS task. Kaiser-Meyer-Olkin value of 0.666 and Bartlett’s Test of Sphericity < 0.05. Parameters with loadings > 0.800 are in bold.

| **Momentum transfer phase Stand-TS** | | | | | | | | |
| --- | --- | --- | --- | --- | --- | --- | --- | --- |
| Principal component | 1 | 2 | 3 | 4 | 5 | 6 | 7 | 8 |
| Variance explained (%) | 30 | 11 | 10 | 7 | 6 | 5 | 4 | 3 |
|  |  |  |  |  |  |  |  |  |
| **Vertical CoM displacement** | **0.862** | -0.054 | -0.005 | 0.013 | -0.090 | 0.150 | 0.329 | 0.059 |
| **Sagittal knee ROM** | **0.808** | -0.010 | -0.005 | 0.120 | 0.039 | 0.083 | 0.453 | -0.031 |
| Transverse knee ROM | 0.760 | 0.034 | 0.018 | 0.045 | -0.096 | -0.195 | 0.005 | 0.039 |
| Sagittal hip ROM | 0.736 | 0.199 | 0.134 | 0.089 | 0.215 | 0.160 | 0.027 | 0.174 |
| Sagittal trunk ROM | 0.696 | 0.199 | 0.177 | 0.159 | 0.222 | 0.232 | -0.247 | 0.071 |
| Frontal knee ROM | 0.632 | 0.133 | 0.433 | 0.168 | -0.142 | 0.042 | -0.120 | 0.228 |
|  |  |  |  |  |  |  |  |  |
| **Frontal ankle velocity range** | -0.091 | **0.875** | 0.026 | 0.145 | 0.013 | 0.200 | -0.045 | 0.092 |
| **Frontal ankle ROM** | 0.035 | **0.844** | -0.021 | 0.189 | 0.040 | -0.034 | 0.134 | 0.117 |
| Sagittal ankle ROM | 0.135 | 0.641 | 0.134 | 0.034 | -0.043 | 0.023 | 0.429 | 0.390 |
| Sagittal ankle velocity range | 0.469 | 0.614 | -0.077 | 0.026 | 0.031 | 0.259 | 0.438 | -0.093 |
| Frontal knee velocity range | 0.210 | 0.576 | 0.323 | 0.291 | 0.180 | 0.222 | -0.147 | 0.162 |
| Transverse ankle velocity range | 0.401 | 0.499 | 0.326 | 0.282 | -0.030 | 0.381 | -0.089 | -0.140 |
| Transverse ankle ROM | 0.441 | 0.471 | 0.105 | 0.353 | -0.166 | -0.120 | 0.325 | -0.122 |
|  |  |  |  |  |  |  |  |  |
| **Frontal hip velocity range** | 0.071 | 0.011 | **0.926** | 0.024 | 0.285 | 0.049 | 0.062 | 0.041 |
| **Transverse knee velocity range** | 0.067 | 0.125 | **0.924** | 0.021 | -0.080 | 0.103 | -0.039 | -0.052 |
| Transverse hip ROM | 0.579 | -0.016 | 0.631 | 0.147 | 0.083 | 0.013 | 0.048 | 0.059 |
|  |  |  |  |  |  |  |  |  |
| Transverse trunk ROM | 0.147 | 0.224 | -0.006 | 0.748 | 0.167 | 0.107 | -0.036 | 0.068 |
| ML CoM velocity | 0.127 | 0.133 | 0.065 | 0.662 | 0.166 | -0.038 | 0.279 | 0.104 |
| Frontal hip ROM | 0.158 | 0.370 | 0.078 | 0.647 | 0.076 | 0.150 | 0.075 | 0.087 |
| Transverse trunk velocity range | -0.112 | -0.311 | -0.059 | 0.504 | 0.058 | 0.472 | 0.056 | 0.239 |
|  |  |  |  |  |  |  |  |  |
| **Sagittal trunk velocity range** | 0.036 | 0.047 | 0.068 | 0.101 | **0.921** | 0.136 | -0.073 | 0.069 |
| **Frontal trunk velocity range** | -0.100 | -0.042 | 0.079 | 0.161 | **0.916** | -0.015 | -0.005 | 0.098 |
| Frontal trunk ROM | 0.453 | 0.126 | 0.118 | 0.478 | 0.556 | 0.061 | -0.053 | 0.009 |
|  |  |  |  |  |  |  |  |  |
| **Vertical CoM velocity** | 0.088 | 0.217 | 0.075 | 0.155 | 0.008 | **0.820** | 0.078 | 0.002 |
| Sagittal knee velocity range | 0.015 | 0.200 | 0.003 | 0.040 | 0.078 | 0.664 | 0.595 | -0.142 |
| Sagittal hip velocity range | 0.330 | 0.091 | 0.383 | 0.021 | 0.287 | 0.627 | -0.256 | 0.198 |
|  |  |  |  |  |  |  |  |  |
| AP CoM velocity | 0.187 | 0.118 | -0.010 | 0.213 | -0.123 | 0.035 | 0.789 | 0.187 |
|  |  |  |  |  |  |  |  |  |
| ML CoM displacement | 0.149 | 0.155 | -0.172 | 0.105 | 0.299 | -0.084 | 0.179 | 0.681 |
| AP CoM displacement | 0.295 | 0.291 | 0.275 | 0.361 | -0.026 | 0.135 | -0.124 | 0.648 |
| Frontal hip velocity range | -0.049 | 0.051 | 0.308 | 0.437 | 0.020 | 0.319 | 0.154 | 0.424 |

**Table S8:** Principal component model of trunk, hip, knee and ankle range of motion (ROM), joint velocity range in sagittal, frontal and transverse planes, and centre of mass (CoM) displacement and velocity range in anteroposterior (AP), mediolateral (ML) and vertical directions during the Momentum transfer phase of Stand-TS task. Kaiser-Meyer-Olkin value of 0.656 and Bartlett’s Test of Sphericity < 0.05. Parameters with loadings > 0.800 are in bold.

| **Extension phase Stand-TS** | | | | |  |  |  |
| --- | --- | --- | --- | --- | --- | --- | --- |
| Principal component | 1 | 2 | 3 | 4 | 5 | 6 | 7 |
| Variance explained (%) | 31 | 16 | 8 | 8 | 6 | 4 | 4 |
|  |  |  |  |  |  |  |  |
| **Sagittal trunk velocity range** | **0.949** | -0.028 | 0.019 | -0.082 | -0.001 | -0.048 | -0.004 |
| **Sagittal hip velocity range** | **0.908** | 0.102 | 0.119 | -0.076 | 0.217 | 0.061 | 0.050 |
| **Frontal trunk velocity range** | **0.882** | 0.346 | -0.072 | -0.148 | 0.037 | 0.109 | 0.026 |
| Sagittal trunk ROM | 0.778 | -0.026 | 0.181 | 0.196 | 0.146 | 0.032 | 0.261 |
| Transverse trunk ROM | 0.687 | 0.220 | 0.177 | -0.018 | -0.018 | 0.329 | 0.277 |
| Transverse trunk velocity range | 0.640 | 0.032 | 0.130 | -0.066 | -0.007 | 0.465 | -0.076 |
|  |  |  |  |  |  |  |  |
| Transverse hip ROM | 0.176 | 0.778 | 0.133 | -0.008 | 0.077 | 0.305 | 0.149 |
| Frontal knee ROM | -0.120 | 0.747 | 0.364 | 0.136 | -0.077 | 0.049 | 0.051 |
| Frontal trunk ROM | 0.363 | 0.694 | -0.031 | 0.013 | 0.059 | 0.017 | 0.264 |
| Transverse knee velocity range | 0.206 | 0.646 | 0.094 | 0.191 | 0.292 | 0.223 | -0.334 |
| Frontal hip velocity range | 0.575 | 0.598 | -0.047 | -0.034 | 0.256 | 0.376 | -0.035 |
| Sagittal knee ROM | -0.038 | 0.525 | -0.182 | 0.388 | 0.178 | -0.057 | 0.408 |
|  |  |  |  |  |  |  |  |
| Sagittal hip ROM | 0.101 | 0.094 | 0.778 | -0.003 | 0.058 | 0.003 | -0.092 |
| Frontal ankle ROM | -0.052 | 0.005 | 0.722 | 0.428 | -0.026 | 0.078 | 0.078 |
| AP CoM displacement | 0.273 | 0.257 | 0.706 | 0.206 | 0.066 | 0.237 | 0.306 |
| AP CoM velocity | 0.128 | 0.179 | 0.647 | 0.103 | 0.416 | 0.315 | 0.066 |
| Vertical CoM displacement | 0.309 | -0.259 | 0.477 | -0.008 | 0.404 | 0.055 | 0.329 |
|  |  |  |  |  |  |  |  |
| **Transverse ankle ROM** | -0.070 | 0.06 | 0.029 | **0.897** | 0.015 | 0.05 | -0.093 |
| **Frontal ankle velocity range** | -0.084 | 0.049 | 0.390 | **0.809** | 0.087 | 0.079 | -0.021 |
| Transverse ankle velocity range | -0.005 | 0.147 | 0.011 | 0.681 | 0.474 | 0.280 | -0.206 |
| Transverse knee ROM | -0.058 | 0.163 | 0.272 | 0.508 | 0.148 | 0.505 | 0.183 |
|  |  |  |  |  |  |  |  |
| **Vertical CoM velocity** | 0.133 | 0.001 | 0.146 | 0.011 | **0.926** | 0.102 | 0.074 |
| **Sagittal knee velocity range** | 0.098 | 0.182 | 0.051 | 0.216 | **0.894** | -0.001 | 0.027 |
|  |  |  |  |  |  |  |  |
| Frontal hip ROM | 0.216 | 0.214 | 0.154 | 0.171 | -0.031 | 0.781 | 0.222 |
| Transverse hip velocity range | 0.522 | 0.180 | 0.104 | 0.073 | 0.308 | 0.653 | -0.138 |
| Frontal knee velocity range | 0.102 | 0.206 | 0.026 | 0.319 | 0.531 | 0.551 | -0.264 |
|  |  |  |  |  |  |  |  |
| ML CoM displacement | 0.203 | 0.231 | 0.249 | -0.014 | 0.031 | 0.088 | 0.648 |
| Sagittal ankle velocity range | -0.033 | 0.062 | 0.350 | 0.408 | 0.426 | -0.017 | -0.584 |
| ML CoM velocity | 0.283 | 0.188 | 0.296 | -0.067 | 0.327 | 0.067 | 0.537 |
| Sagittal ankle ROM | 0.108 | -0.002 | 0.462 | 0.459 | 0.130 | 0.027 | -0.536 |
